# Dietary supplementation with arachidonic acid increases arachidonic acid content in paw, but does not affect arthritis severity or prostaglandin E_2_ content in rat adjuvant-induced arthritis model

**DOI:** 10.1186/1476-511X-14-3

**Published:** 2015-01-16

**Authors:** Norifumi Tateishi, Yoshihisa Kaneda, Saki Kakutani, Hiroshi Kawashima, Hiroshi Shibata, Ikuo Morita

**Affiliations:** Institute for Health Care Science, Suntory Wellness Ltd., 1-1-1 Wakayamadai, Shimamoto, Osaka, 6188503 Japan; Department of physiology and pharmacology, School of advanced science and engineering, Waseda University, Tokyo, Japan; Department of Cellular Physiological Chemistry, Graduate School, Tokyo Medical and Dental University, Tokyo, Japan

**Keywords:** Arachidonic acid, Arthritis, Prostaglandin E_2_, Lipoxin A_4_

## Abstract

**Background:**

Arachidonic acid (ARA) is an essential fatty acid and a major constituent of biomembranes. It is converted into various lipid mediators, such as prostaglandin E_2_ (PGE_2_), which is involved in the development of rheumatoid arthritis (RA). However, the effects of dietary ARA on RA are unclear. Our objective was to clarify the effects of dietary ARA on an experimental rat arthritis model.

**Methods:**

Lew rats were fed three contents of ARA diet (0.07%, 0.15% or 0.32% ARA in diet (w/w)), a docosahexaenoic acid (DHA) diet (0.32% DHA), or a control diet. After 4 weeks, arthritis was induced by injection of Freund’s complete adjuvant into the hind footpad. We observed the development of arthritis for another 4 weeks, and evaluated arthritis severity, fatty acid and lipid mediator contents in the paw, and expression of genes related to lipid mediator formation and inflammatory cytokines. Treatment with indomethacin was also evaluated.

**Results:**

The ARA content of phospholipids in the paw was significantly elevated with dietary ARA in a dose-dependent manner. Dietary ARA as well as DHA did not affect arthritis severity (paw edema, arthritis score, and bone erosion). PGE_2_ content in the paw was increased by arthritis induction, but was not modified by dietary ARA. Dietary ARA did not affect the contents of other lipid mediators and gene expression of cyclooxygenase (COX)-1, COX-2, lipoxgenases and inflammatory cytokines. Indomethacin suppressed arthritis severity and PGE_2_ content in the paw.

**Conclusion:**

These results suggest that dietary ARA increases ARA content in the paw, but has no effect on arthritis severity and PGE_2_ content of the paw in a rat arthritis model.

**Electronic supplementary material:**

The online version of this article (doi:10.1186/1476-511X-14-3) contains supplementary material, which is available to authorized users.

## Introduction

Polyunsaturated fatty acids (PUFAs), such as arachidonic acid (ARA) and docosahexaenoic acid (DHA), are natural nutrients present in common foodstuffs (e.g., egg yolk, meat, and fish oil) and are physiologically important constituents of biomembranes. ARA is an n-6 fatty acid and is converted from Linoleic acid (LA) in vivo. ARA also acts as the substrate for various lipid mediators, such as prostaglandins (PGs), leukotrienes, lipoxins (LX), endocannabinoids, and epoxyeicosatetranoates [1–3]. The recent studies demonstrated that the conversion ability from LA to ARA was decreased with aging [4] and that dietary ARA supplementation improved cognitive response [5, 6] and cardiovascular function [7, 8] in the elderly people and aged rats.

It has been clarified that dietary ARA affects the ARA content of phospholipids in humans and animals [9–14]. An ex vivo study using cells prepared from animals in which dietary fatty acid intake was manipulated showed that different diets impacted the production of PGs and leukotrienes [15–18]. We reported that ARA supplementation in healthy Japanese elderly individuals increased plasma ARA but did not increase ARA-derived lipid mediators or clinical parameters, including inflammatory parameters such as C-reactive protein, interleukin-6 (IL-6), and tumor necrosis factor-α (TNF-α) [12]. We recently studied the effects of dietary ARA on acute inflammation and reported that dietary ARA increased the ARA and LXA_4_ contents in the colon but did not affect the severity of inflammation or PGE_2_ content in a murine colitis model [14].

Rheumatoid arthritis (RA) is one of the major autoimmune diseases and is associated with chronic inflammation of the joints and bones. Biological disease-modifying antirheumatic drugs are frequently recommended for RA therapy, indicating that inflammatory cytokines are important molecules in the pathology of RA [19–21]. Non-steroidal anti-inflammatory drugs (NSAIDs) and cyclooxygenase (COX) inhibitors are no longer first-line drugs for RA, but before the advent of biologic therapy, these agents were widely used for RA because lipid mediators produced from ARA by COX, such as PGE_2_, are involved in the development of RA [22, 23]. Therefore, ARA metabolism is still important in the treatment of RA, but the effects of dietary ARA on chronic inflammation, such as RA, are not fully understood.

In the present study, to clarify the effects of dietary ARA on chronic inflammation and PGE_2_ status, we evaluated the effects of ARA at various doses on the severity of an adjuvant-induced arthritis (AIA) model in rats, and determined the contents of ARA and ARA-derived lipid mediators and the expression of genes related to these lipid mediators and inflammatory cytokines.

## Materials and methods

### Animals, diets, and experimental design

Experiments were approved by the Animal Care and Use Committee of Suntory Holdings Ltd. (Osaka, Japan), and we followed the Guidelines for Animal Care and Use of Suntory Holdings Ltd. Seventy 4-week-old male Lew rats were obtained from Charles River Japan (Yokohama, Japan). Rats were housed under standard conditions and had free access to water and diet.

We used five types of diet based on the AIN-76 rodent diet (Table 1). The AIN-76 contains 5% fat in diet and the composition of the diets was the same as that of AIN-76, except for lipids in this study. In brief, AIN-76 rodent diet was composed of 20% casein, 43% cornstarch, 12% alpha-type cornstarch, 10% sucrose, 5% fat, 5% cellulose fiber, 3.5% AIN-76 salt mix, 1.0% AIN-76 vitamin mix, 0.3% dl-methionine and 0.2% choline bitartrate. The lipids used in the present study were ARA-enriched triacylglycerol (SUNTGA40S) (lot no. 100120A1; Nippon Suisan Kaisha Ltd., Tokyo, Japan), fish oil (DHA-27 W) (lot no. 1003251; Maruha Nichiro Food Corporation Ltd., Tokyo, Japan), and palm oil, soybean oil, and linseed oil (Showa Kousan Ltd., Osaka, Japan). The ARA content was approximately 42.5% in ARA-enriched triacylglycerol, which was used in diets for the low dose of ARA (ARA(L)), the medium dose of ARA (ARA(M)), and the high dose of ARA (ARA(H)) groups. DHA and eicosapentaenoic acid (EPA) contents were approximately 26.8 % and 6.8%, respectively, in fish oil, which was used in the diet for the DHA groups. Control diet (CON) had no ARA, DHA and EPA. Palm oil, soybean oil, and linseed oil contained no ARA, DHA, or EPA, and were used to adjust the amounts of total lipids, total saturated fatty acids (S), total monounsaturated fatty acids (M), total polyunsaturated fatty acids (P), total n-6 fatty acids, and total n-3 fatty acids to similar levels in the five diets, respectively (Table 1). Experimental diets were stored at 4°C and protected from light to prevent oxidation.

**Table 1:** **Fatty acid composition of the diets**

Fatty acids	CON	ARA(L)	ARA(M)	ARA(H)	DHA
		g/100 g fatty acids			
16:0 palmitic acid	27.1	28.0	28.1	27.4	28.3
18:0 stearic acid	4.4	4.7	4.8	5.1	4.6
18:1(n-9) oleic acid	32.0	32.2	31.4	29.5	30.7
18:2(n-6) linoleic acid	23.4	20.9	19.1	15.8	21.3
18:3(n-3) α-linolenic acid	11.6	10.8	10.9	11.6	2.5
20:3(n-6) dihomo-γ-linolenic acid	0.0	0.1	0.3	0.6	0.0
20:4(n-6) arachidonic acid	0.0	1.4	2.9	6.3	0.5
20:5(n-3) eicosapentaenoic acid	0.0	0.0	0.0	0.0	1.6
22:6(n-3) docosahexaenoic acid	0.0	0.0	0.0	0.0	6.3
Others	1.5	1.9	2.5	3.7	4.2
Total	100	100	100	100	100
PUFA	35.7	34.0	34.1	35.5	33.4
MUFA	32.6	32.6	31.9	30.0	33.6
SFA	31.8	33.4	34.1	34.5	33.0
n-6/n-3	2.0	2.1	2.0	2.0	2.2

Fatty acid composition of the experimental diets. Numbers in the table are expressed in percent except for the n-6/n-3 ratio. PUFA: polyunsaturated fatty acid; MUFA: monounsaturated fatty acid; SFA: saturated fatty acid. Note that each diet comprised equa.

### Induction and evaluation of AIA

**Figure 1:**
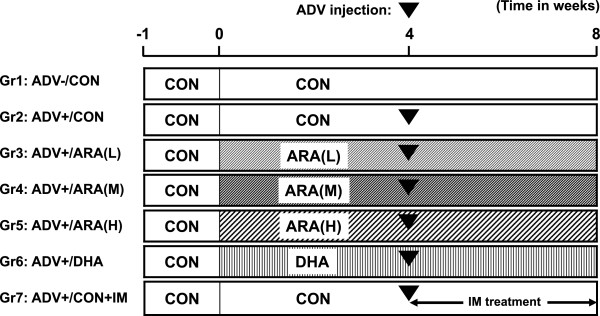
**Experimental procedure for rat adjuvant-induced arthritis model in the present study.**

After a 1-week acclimation period, rats were randomly assigned to seven groups of 10 rats according to body weight, and received one of the five diets described above for 8 weeks (Figure 1). Arthritis was induced by subcutaneous injection of 0.05 mg of *Mycobacterium butyricum* (Difco Laboratories Inc., Detroit, MI) suspended in 0.1 mL of liquid paraffin into the left hind footpad on day 29 (day 1 was defined as the day when dietary manipulation started). The dose of *M. butyricum* was designed to induce a moderate degree of arthritis for detecting both the promotion and suppression of arthritis by diet or indomethacin (Additional file 1: Table S1). Liquid paraffin without *M. butyricum* was injected in group 1. Indomethacin (1 mg/kg body weight) suspended in 0.1% carboxymethyl cellulose was orally administered once daily from day 29 to day 56 in group 7. Body weight and hind-paw swelling were measured intermittently. The volumes of the ipsilateral (left) and contralateral (right) hind paws were measured using a plethysmometer (Unicom, Chiba, Japan) before adjuvant injection (day 29) and on days 36, 43, 50, and 57. The severity of arthritis symptoms in the whole body was graded according to Yamaguchi et al. [24], with minor modification. In brief, the clinical severity of arthritis was scored as follows: 0 – normal; 1 – very slight; 2 – slight; 3 – moderate; 4 – marked and 5 – strong. The cumulative clinical arthritis score per rat was the total of five individual scores for ears, tail, forelimbs, ipsilateral hind paw and contralateral hind paw, with a maximum score of 25. At the end of the experiments, rats were anesthetized with isoflurane and killed via blood sampling from the abdominal aorta and exsanguination. Knee and ankle joints of the contralateral paw were fixed with 10% neutral buffered formalin and used for radiography analysis. The X-ray score was defined according to Fukawa et al. [25] as the total score of a combination of osteopenia, bone erosion, and new bone formation as follows: 0 – no change; 1 – slight change, 2 – moderate change; and 3 – severe change. The ipsiralateral hind paw was immediately frozen in liquid nitrogen and used for analyses of fatty acids, lipid mediators, and gene expression.

### Fatty acid analysis

Lipids in the diets, paws, and plasma were extracted and purified by the method of Folch et al. [26]. Lipids in the paw and plasma were separated into phospholipids (PL) and other lipid fractions by thin-layer chromatography using silica gel 60 (Merck, Darmstadt, Germany). The solvent system consisted of hexane/diethyl ether (7/3, v/v). Fatty acid residues in extracted lipids or separated phospholipids were analyzed by the method of Sakuradani et al. [27]. Briefly, each lipid fraction was incubated with an internal standard (pentadecanoic acid) in methanolic HCl at 50°C for 3 h to transmethylate fatty acid residues to fatty acid methyl esters, which were extracted with *n*-hexane and analyzed by capillary gas–liquid chromatography.

### Analysis of lipid mediators of footpad homogenates

PGE_2_, PGE_2_-d4, leukotriene B_4_ (LTB_4_), LTB_4_-d4, LXA_4_, and LXA_4_-d5 were obtained from Cayman Chemical (Ann Arbor, MI). The methods for extraction and analysis of lipid mediators were reported previously [28, 29]. Briefly, footpad tissue frozen in liquid nitrogen was ground using a Multi-Beads Shocker MB701(S) (Yasui Kikai, Osaka, Japan) and homogenized with ice-cold ethanol. A fixed amount of PGE_2_-d4, LTB_4_-d4, and LXA_4_-d5 was added to all homogenates as an internal standard. After centrifugation, each supernatant was dried by centrifugal evaporation, and residues were dissolved in methanol, washed, and concentrated with SPE cartridges (Empore disk cartridge C18 SD; 3 M, St. Paul, MN). An Agilent 1200 HPLC system (Agilent Technologies, Santa Clara, CA) equipped with a Cadenza CD-C18 column (3 mm, 2 mm i.d. × 150 mm; Imtakt, Kyoto, Japan) and quadruple linear ion trap hybrid mass spectrometer, 4000 Q TRAP, with an electrospray interface (Applied Biosystems/MDS SCIEX, Concord, Canada) was used for quantification. The mass spectrometer was operated in negative ion mode with selected reaction monitoring. PGE_2_ and PGE_2_-d4 were detected by monitoring mass transitions at *m/z* 351 → 271 for PGE_2_ and *m/z* 355 → 275 for PGE_2_-d4 at a collision energy of −24 V. The quantitative range of PGE_2_ was 0.3 – 100 ng/injection. LTB_4_ and LTB_4_-d4 were detected by monitoring mass transitions at *m/z* 335 → 195 for LTB_4_ and *m/z* 339 → 197 for LTB_4_-d4 at a collision energy of −24 V. The quantitative range of LTB_4_ was 0.6 – 200 pg/injection. LXA_4_ and LXA_4_-d5 were detected by monitoring mass transitions at *m/z* 351 → 115 for LXA_4_ and *m/z* 356 → 115 for LXA_4_-d5 at a collision energy of −22 V. The quantitative range of LXA_4_ was 3 – 1000 pg/injection.

### Quantitative real-time polymerase chain reaction (QRT-PCR)

The methods for QRT-PCR were as reported previously [30], with some modification. In brief, the total RNA from hind-footpad tissues stored at −80°C was extracted using Isogen (Nippon Gene Co., Ltd., Toyama, Japan) and purified with an RNeasy mini kit (Qiagen GmbH, Hilden, Germany). The total RNA (2.0 *μ*g) was reverse-transcribed with random primers using High-Capacity cDNA Reverse Transcription Kits (Applied Biosystems, Foster City, CA) in accordance with the recommendations of the manufacturer. To quantify the gene expression, cDNA was amplified for various gene targets by QRT-PCR using the ABI PRISM 7900 Sequence Detection System (Applied Biosystems). All primers and probes used were purchased as TaqMan Gene Expression Assays: cytosolic phospholipase A2 (cPLA2, Rn00591916_m1), COX-1 (Rn00566881_m1), COX-2 (Rn01483828_m1), arachidonate 5-lipoxygenase (5-LOX, Rn00563172_m1), arachidonate 12/15-lipoxygenase (12/15-LOX, Rn00696151_m1), TNF-α (Rn01525859_g1), IL-1beta (Rn0058432_m1), IL-6 (Rn01410330_m1) and IL-10 (Rn00566881_m1) (Applied Biosystems). PCR results were analyzed with ABI SDS software (Applied Biosystems). Relative expression levels of the genes in each sample were determined by the Comparative Ct Method. Expression assays for each gene were normalized against glyceraldehyde-3-phosphate dehydrogenase (GAPDH, Rn01775763_g1) and expressed as fold change relative to that of the disease-control group (group 2).

### Statistical analysis

Data are presented as means ± SD. Data were analyzed by the unpaired two-tailed t test or one-way analysis of variance followed by Dunnett or Steel multiple comparisons. Correlation analyses were performed using the Spearman correlation test. P < 0.05 was considered statistically significant.

## Results

### Fatty acid content of the hind-paw and plasma

**Figure 2:**
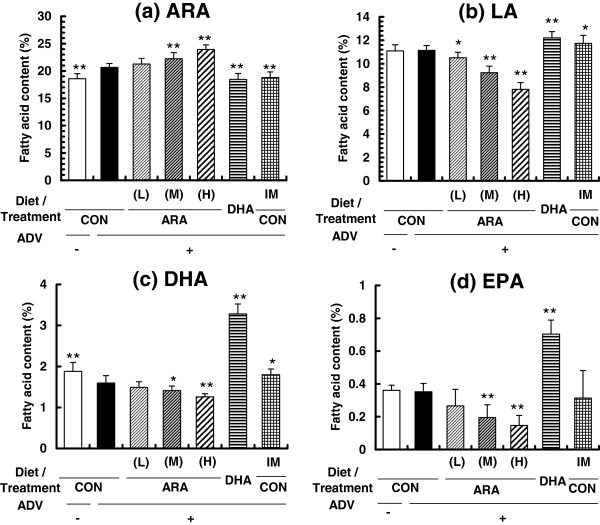
**Fatty acid composition of phospholipids ((a) ARA, (b) LA, (c) DHA, and (d) EPA) in the ipsilateral hind paw from adjuvant-induced arthritis rats.** Data are means ± SD. *p < 0.05, **p < 0.01 versus ADV+/CON diet group (n = 10 per group).

Figure 2 shows the fatty acid content of the phospholipids of the ipsilateral hind-paw. ARA was significantly higher and DHA was significantly lower in paws of the adjuvant-treated CON diet group (ADV+/CON, group 2) compared with those of the adjuvant-untreated CON diet group (ADV-/CON, group 1) (Figure 2a). Dietary ARA significantly increased the ARA content of paws in a dose-dependent manner. The ARA content in the ADV+/CON and ADV+/ARA(H) (group 5) groups was 20.7 ± 0.7% and 23.9 ± 0.9%, respectively. Dietary ARA inversely decreased linoleic acid (LA), DHA, and EPA contents of paws in an ARA dose-dependent manner (Figure 2b, c, d). In the ADV+/DHA group (group 6), the DHA content was high (3.3 ± 0.2%) and the ARA content was low (18.5 ± 1.1%) compared to the ADV+/CON group (DHA content, 1.6 ± 0.2%; and ARA content, 20.7 ± 0.7%). In the adjuvant- and indomethacin- treated CON diet group (ADV+/CON + IM group, group 7), ARA was lower and LA and DHA were higher in paws compared with those of the ADV+/CON group. A similar tendency was observed for plasma phospholipids (Additional file 2: Figure S1a, b, c, d). Dietary ARA significantly increased the ARA content of plasma in a dose-dependent manner. Total n-6 and n-3 contents and n-6/n-3 ratio in phospholipids of paws and plasma are shown in Additional file 3: Figure S2a-f. In the paw, total n-3 was lower and n-6/n-3 ratio was higher in the ARA diet groups, and total n-3 was higher and total n-6 and n-6/n-3 ratio was lower in the DHA diet group compared with ADV+/CON group. Similar tendency was observed in plasma.

### Effects of dietary ARA on AIA symptoms

**Figure 3:**
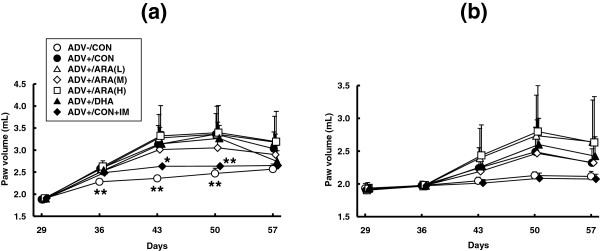
**Time course of adjuvant-induced paw edema in ipsilateral (a) and contralateral hind paws (b).** Rats were fed three types of ARA diet (ARA(L), ARA(M) or ARA(H)), DHA diet, or control diet (CON) from day 0. Adjuvant arthritis was induced by subcutaneous injection of adjuvant containing 0.05 mg of *M. butyricum* (ADV+) or without *M. butyricum* (ADV-) on day 29. Rats in the IM group were orally administered indomethacin (1mgkg^−1^) once a daily from day 29 to day 56. Data are means ± SD. *p < 0.05, **p < 0.01 versus ADV+/CON diet group (n = 10 per group).

**Figure 4:**
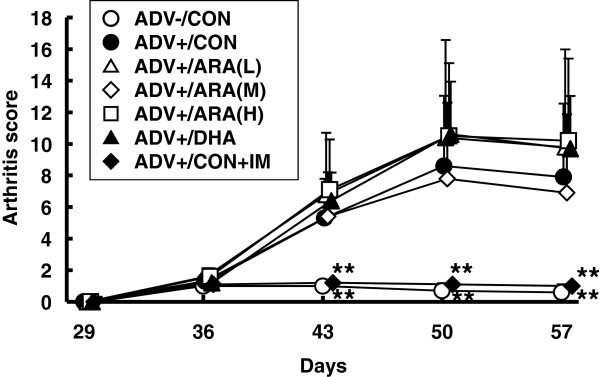
**Time course of the arthritis score.** The score was determined as described in Materials and methods. Data are means ± SD. **p < 0.01 versus ADV+/CON diet group (n = 10 per group).

**Figure 5:**
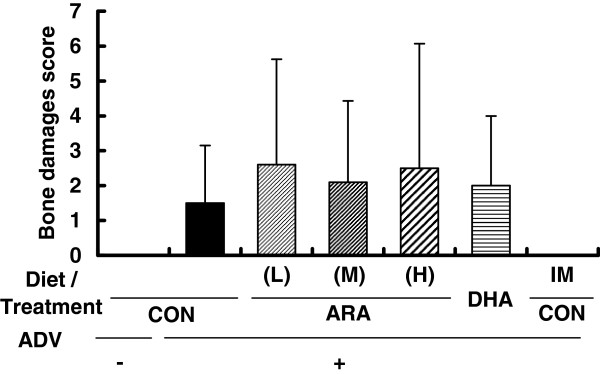
**Bone erosion score for the joint of the contralateral hind paws from adjuvant-induced arthritis rats.** At the end of the experiments, knee and ankle joints of the contralateral paw were fixed with 10% neutral buffered formalin and X-ray score was defined as described in Materials and Methods. Data are means ± SD (n = 10 per group).

Body weight was not different between groups before AIA induction, but was higher in the ADV-/CON and ADV+/CON + IM groups than in the ADV+/CON group on days 50 and 57. Dietary ARA or DHA did not affect body weight through the experiment (Additional file 4: Figure S3). Adjuvant injection induced a moderate and significant paw edema in the ipsilateral side (on day 50: the ADV+/CON group, 3.4 ± 0.5 ml; and the ADV-/CON group; 2.5 ± 0.1 ml) (Figure 3a). The volumes of ipsilateral paws in the groups fed ARA or DHA were not different from those in the ADV+/CON group. The volume of ipsilateral paws was significantly suppressed in the ADV+/CON + IM group. Adjuvant injection also seemed to induce moderate paw edema in the contralateral side (on day 50: the ADV+/CON group, 2.5 ± 0.4 ml; the ADV-/CON group, 2.1 ± 0.1 ml), but differences were not significant (Figure 3b). No effects of diets or indomethacin were observed in contralateral paw volume.The systemic arthritis score was significantly increased after arthritis induction and reached 8.6 ± 4.5 on day 50 in the ADV+/CON group (0.7 ± 0.5 in the ADV-/CON group) (Figure 4). Arthritis scores in the groups fed ARA or DHA were not different from those in the ADV+/CON group. Arthritis scores were significantly suppressed in the ADV+/CON + IM group. Moderate bone damage seemed to be induced (the ADV+/CON group, 1.5 ± 1.7; and the ADV-/CON group, 0), but differences were not significant (Figure 5). No effects of diets or indomethacin were observed in bone damage score.

### Lipid mediator and gene expression

**Figure 6:**
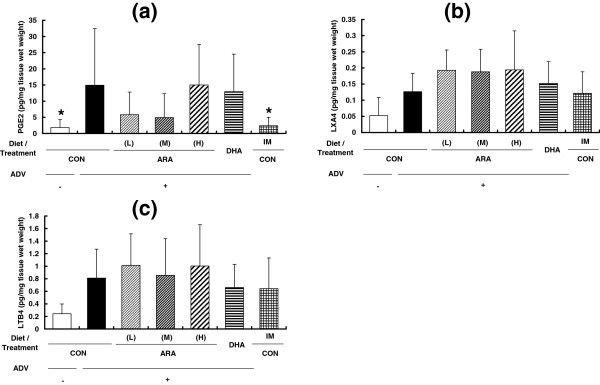
**Prostaglandin E**_**2**_ **(PGE**_**2**_ **), lipoxin A4 (LXA**_**4**_ **), and leukotriene B4 (LTB**_**4**_ **) levels in the ipsilateral hind paw from adjuvant-induced arthritis rats. a**; PGE_2_, **b**; LXA_4_, **c**; LTB_4_. Data are means ± SD. *p < 0.05 versus ADV+/CON diet group (n = 10 per group).

**Figure 7:**
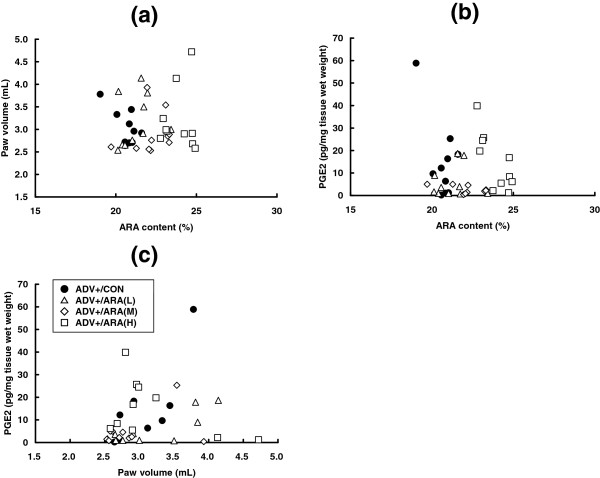
**Correlation analysis.** Correlation analyses were performed between the ARA content of the ipsilateral hind paw versus paw edema (disease marker) **(a)**, PGE2 content **(b)**, and between paw edema versus PGE2 contents **(c)**. ●; ADV+/CON, △; ADV+/ARA(L), ◇; ADV+/ARA(M), ☐; ADV+/ARA(H).

Arthritis induction significantly increased the PGE_2_ content of the paw (the ADV+/CON group, 15.0 ± 17.5 pg/mg tissue wet weight; and the ADV-/CON group; 1.8 ± 2.5 pg/mg tissue wet weight) (Figure 6a). The PGE_2_ content of paws in groups fed ARA or DHA were not different from that in the ADV+/CON group. Indomethacin treatment significantly suppressed the PGE_2_ content of the paw. The content of LXA_4_ (the ADV+/CON group; 0.13 ± 0.1 pg/mg tissue wet weight; and the ADV-/CON group, 0.05 ± 0.1 pg/mg tissue wet weight) and LTB_4_ (the ADV+/CON group; 0.81 ± 0.46 pg/mg tissue wet weight; and the ADV-/CON group, 0.24 ± 0.16 pg/mg tissue wet weight) seemed to increase with arthritis induction, but not significantly (Figure 6b and c). No effects of diets or indomethacin were observed in the contents of LXA_4_ and LTB_4_. To elucidate the relationship between the ARA content and PGE_2_ content in the paw and paw volume, we analyzed the correlation of these parameters in the adjuvant- and ARA-treated groups (groups 2–5). As shown in Figure 7, we detected no correlation of ipsiralateral paw volume or paw PGE_2_ content with the ARA content in the paw (Figure 7a and b), paw PGE_2_ content with ipsiralateral paw volume (Figure 7c).

We assayed the expression of genes related to lipid mediator formation, such as cPLA2, COX-1, COX-2, 5-LOX, and 12/15-LOX (Additional file 5: Figure S4 a-e). Only COX-1 expression in the ADV-/CON group was significantly lower than that in the ADV+/CON group, but no other gene expressions differed between the ADV- and ADV+/CON groups. The ARA or DHA diet had no effect on these gene expressions. Indomethacin treatment increased only ALOX5 gene expression. In addition, to determine the effects of diets on cytokine production related to inflammation, we also analyzed gene expression of IL-1beta, IL-6, TNF-α, and IL-10. There were no differences between these groups in these genes (Additional file 6: Figure S5 a-d).

## Discussion

In the present study, dietary ARA supplementation significantly increased the ARA content in the inflamed paw in a dose-dependent manner, but did not affect the severity and content of lipid mediators in an AIA rat model. This is the first study to demonstrate the effect of dietary ARA on AIA in rats.

Some studies have reported that dietary n-6 fatty acids, mainly LA, exacerbated the symptoms of arthritis models compared to n-3 fatty acids [31–34]. It might be believed that dietary ARA also exacerbates arthritis because ARA is the major n-6 PUFA in the body and is converted to various proinflammatory lipid mediators. However, it remains unknown whether dietary ARA itself exacerbates arthritis or not. To detect the effect of ARA accurately, we carefully designed the experimental conditions and adjusted the severity of AIA to moderate levels (Additional file 1: Table S1). Therefore we could detect both suppression and exacerbation of AIA if ARA had such potential and aimed to prevent a false-negative misjudgement of the potential of ARA. In the present study, dietary ARA significantly increased ARA in phospholipids of the inflamed paw and plasma in a dose-dependent manner in AIA (Figure 2 and Additional file 2: Figure S1), which is consistent with previous studies in humans [9–12] and animals [13–15]. Despite local and systemic increases in ARA, dietary ARA did not exacerbate any AIA parameters, such as paw edema, arthritis score, or bone erosion. Our data in this study are supported by the previous papers. Severity of murine colitis, a typical acute bowel inflammatory model, was also unchanged by dietary ARA [14]. Conversely, there was a report that ARA ethyl ester improved some parameters (body weight loss and diarrhea) in a similar colitis model [35]. A human study revealed that dietary ARA did not affect inflammatory parameters (plasma CRP, IL-6, and TNF-α) in healthy participants [12]. Ultimately, there has been a lack of information regarding whether dietary ARA exacerbates the severity of the inflammatory diseases. The present results suggest that dietary ARA did not affect chronic inflammatory diseases like arthritis, as well as acute inflammatory diseases in previous studies mentioned above. Furthermore, interestingly, ARA content in ADV+/CON group was higher and DHA content in this group was lower compared with ADV-/CON group (Figure 2). Similar tendency was observed in plasma (Additional file 2: Figure S1). Total n-6 and n-3 contents also showed the similar tendency (Additional file 3: Figure S2). The animals in both groups have been fed the same diet (CON) without either ARA or DHA, suggesting that some kind of ARA and DHA metabolism might be modified by chronic inflammation. The reason is unclear at this stage.

Considering the reason why dietary ARA did not affect AIA severity, it is important to examine the changes in lipid mediators from ARA. This is the first study to demonstrate a relationship between ARA composition and ARA-derived lipid mediators in an AIA model. In particular, PGE_2_ is well known to be a proinflammatory cytokine for various types of inflammation and a key factor in RA. In fact, NSAIDs have been one of the important pharmaceutical treatments against RA due to their suppression of PGE_2_ formation through COX inhibition [1, 22, 23]. The COX-PGE_2_ axis dependency was confirmed also in the present results, that is, an 8-time elevation of paw PGE_2_ content was evoked by arthritis induction, and indomethacin treatment clearly suppressed PGE_2_ production and AIA parameters (Figure 6). Surprisingly, the PGE_2_ content in the paw was not increased in either the ARA(L), ARA(M), or ARA(H) groups, although the ARA content in the paw was significantly increased in a dose-dependent manner (Figure 2). These findings may seem unreasonable because ARA is a precursor of PGE_2_. However, in the previous studies, dietary ARA did not affect the content of PGE_2_ and/or its metabolites in serum and urine in healthy human participants [12] and in the colon of a murine colitis model [14]. Taken together, it is suggested that dietary ARA does not affect the PGE_2_ content in vivo, and that lack of elevation of the PGE_2_ content is one of the reasons for no change in arthritis severity. These hypotheses are also supported by the correlation results for PGE_2_ and arthritis parameters (Figure 7), and seem to be different to the results of previous ex vivo or in vitro studies which indicated an increase in eicosanoid production with ARA level [15–17, 36]. The reasons for these differences are unclear, but may be related to the fact that intensive and unphysiological stimuli are used in ex vivo or in vitro experiments. PGE_2_ metabolism may be also involved. Ex vivo or in vitro experimental conditions for PGE_2_ production may need to be carefully considered to understand in vivo situations.

Another ARA-derived lipid mediator, LXA_4_, is produced by 15- and 5-lipoxygenases and is clarified to have an anti-inflammatory role [37]. In the animal arthritis model, 12/15-lipoxigenase–deficient mice showed enhanced inflammatory effects and decreased levels of LXA_4_[38], and a lipoxin receptor agonist could modulate the immune response and reduce the severity of murine arthritis [39]. In the present study, the LXA_4_ content in the ARA groups was slightly higher compared with the control group but no significant differences or dose-dependency was found (Figure 6), although dietary ARA increased the LXA_4_ content in the colon in our previous study using a murine colitis model [14]. These results suggest that the effects of dietary ARA on LXA_4_ production differ depending on the inflammation model. LTB_4_, one of the major leukotrienes, was shown to be produced in the joints of RA patients [40, 41]. In the present study, the LTB_4_ content in the paw tended to be increased by arthritis induction and were not affected by dietary ARA. These results are similar to those for the colitis model [14], and suggest that dietary ARA has little effect on LTB_4_ production.

Gene expression of COX-1 and COX-2 is related to PGE_2_ synthesis, that of 15-LOX and 5-LOX is related to LXA_4_ and LTB_4_ synthesis, and that of cPLA_2_ is related to all of these mediators through release of ARA from membrane phospholipids. Gene expression of these mediators was unchanged by dietary ARA (Additional file 5: Figure S4). This is consistent with the results showing that the PGE_2_, LXA_4_, and LTB_4_ contents were unchanged by dietary ARA (Figure 6). However, because we could not observe increases in gene expression by arthritis induction except for COX-1, we cannot draw a firm conclusion about its effects on gene expression. Further studies, for example a time course experiment [42, 43], are necessary to determine the effects of dietary ARA on gene expression related to lipid mediators. These results are similar to the case of gene expression of inflammatory cytokines. In the present study, dietary ARA did not affect the gene expressions of these cytokines in vivo (Additional file 6: Figure S5). However, we could not observe increases of gene expression by arthritis induction, and further studies are needed to determine the effect of dietary ARA on gene expression of inflammatory cytokines.

In this study, we showed the amounts of lipid mediators in paws, but their contribution to the disease state is not fully confirmed. Although PGE_2_, LXA_4_ and LTB_4_ are known to be important as described above, it remains unclear whether their contents in the present experiment are enough to show physiological/pathological activities or not. It may be revealed by local injection of PGE_2_, LXA_4_ and LTB_4_ or the specific antagonists for example. The amount of lipid peroxides, free radical generation and anti-oxidant content in the tissues and cells might help the clarification. Furthermore, it has been well-known that lipid mediators might affect the amount of TGF-β beta [44]. The cytokine profile is also expected to be clarified by further studies.

DHA, EPA, or fish oil was reported to be effective against arthritis in both animal and clinical studies [31–34, 45], while some studies failed to show any benefit [32, 46]. In the present study, DHA administration significantly increased the contents of DHA and EPA in the paw, but did not affect either inflammatory parameters or the amounts of PGE_2_, LXA_4_, and LTB_4_. One of the reasons may be that the experimental diets in the present study were designed to have similar n-6/n-3 ratio, and that therefore n-6/n-3 ratio was almost the same in the control (2.0) and DHA (2.2) diets. The n-6/n-3 ratios in the previous studies seem different between the control and DHA, EPA or fish oil groups or unclear [31–34], and it might cause the difference of DHA efficacy.

To the best of our knowledge, there is one study in 1997 regarding the effects of ARA on AIA in essential fatty acid-deficient (EFAD) rats. ARA supplementation could increase the ARA content in EFAD and recover the suppressed inflammatory response in EFAD rats compared with normal rats [47]. This was an early and important report, but it does not necessarily reflect the situation for general arthritis. The fatty acid profile in EFAD is quite abnormal, that is, ARA and LA are extremely reduced. Furthermore, Mead acid, which was clarified to have anti-inflammatory properties [48–50], was endogenously synthesized instead of ARA in EFAD and was reduced by ARA administration [47]. It is also reported that Mead acid is detected in articular cartilage of newborn animals, suggesting that Mead acid might have unknown roles in cartilage development [51]. Therefore, it is difficult to estimate the effects of ARA on AIA from the previous report under the EFAD condition.

Dietary ARA intakes for the ARA(L), ARA(M), and ARA(H) groups were estimated to be approximately 35, 73, and 156 mg/kg/day, respectively, on the assumption that rats consumed a diet about 10% of their body weight daily. In humans, the average ARA intake from daily foods is approximately 150–200 mg ARA per day [52]. Compared to the ARA intake of humans, the ARA doses used in the present study are markedly higher. The results in the present study may thus be regarded as those under excess ARA administration.

## Conclusion

Dietary ARA supplementation significantly increased the content of ARA in the inflamed paw in a dose-dependent manner, but did not affect arthritis inflammatory parameters or the content of lipid mediators in an AIA rat model.

## Electronic supplementary material

Additional file 1: Table S1: Arthritis induction by various doses of *M. butyricum.* (XLS 18 KB)

Additional file 2: Figure S1: Fatty acid content of phospholipids ((a) ARA, (b) LA, (c) DHA, and (d) EPA) in the plasma from adjuvant-induced arthritis rats. Data are means ± SD. *p < 0.05, **p < 0.01 versus ADV+/CON diet group (n = 10 per group). (PPT 74 KB)

Additional file 3: Figure S2: Total n-6, total n-3 and n-6/n-3 ratio on fatty acid content of phospholipids in the paw (a, b, c) or plasma (d, e, f) from adjuvant-induced arthritis rats. Data are means ± SD. *p < 0.05, **p < 0.01 versus ADV+/CON diet group (n = 10 per group). (PPT 77 KB)

Additional file 4: Figure S3: Body weight change before and after arthritis induction. Rats were fed three types of ARA diet (ARA(L), ARA(M), or ARA(H)), DHA diet, or control diet (CON) from day 0. Adjuvant arthritis was induced by subcutaneous injection of adjuvant containing 0.05 mg of *M. butyricum* (ADV+) or without *M. butyricum* (ADV-) on day 29. The IM group rats were orally administered indomethacin (1mgkg^−1^) once daily from day 29 to day 56. Data are means ± SD. **p < 0.01 versus ADV+/CON diet group (n = 10 per group). (PPT 75 KB)

Additional file 5: Figure S4: Expression of genes related to lipid mediator formation. cPLA2 (a), COX-1 (b), COX-2 (c), 5-LOX (d) and 15-LOX (e) in the ipsilateral hind paw from adjuvant-induced arthritis rats fed CON, ARA(L), ARA(M), ARA(H) and DHA diet. Data are means ± SD. **p < 0.01 versus ADV+/CON diet group (n = 10 per group). (PPT 88 KB)

Additional file 6: Figure S5: Expression of genes related to inflammation. IL-1beta (a), IL-6 (b), TNF-α (c), and IL-10 (d) in the ipsilateral hind paw from adjuvant-induced arthritis rats fed a CON, ARA(L), ARA(M), ARA(H), and DHA diet (n = 10 per group). Data are means ± SD. (PPT 75 KB)
