# Supplementary figures and images for: Dietary supplementation with arachidonic acid increases arachidonic acid content in paw, but does not affect arthritis severity or prostaglandin E2 content in rat adjuvant-induced arthritis model
Source: Lipids Health Dis. 2015 Jan 16;14:3. doi: 10.1186/1476-511X-14-3 (PMC4417218; doi:10.1186/1476-511X-14-3)

## Slide 1
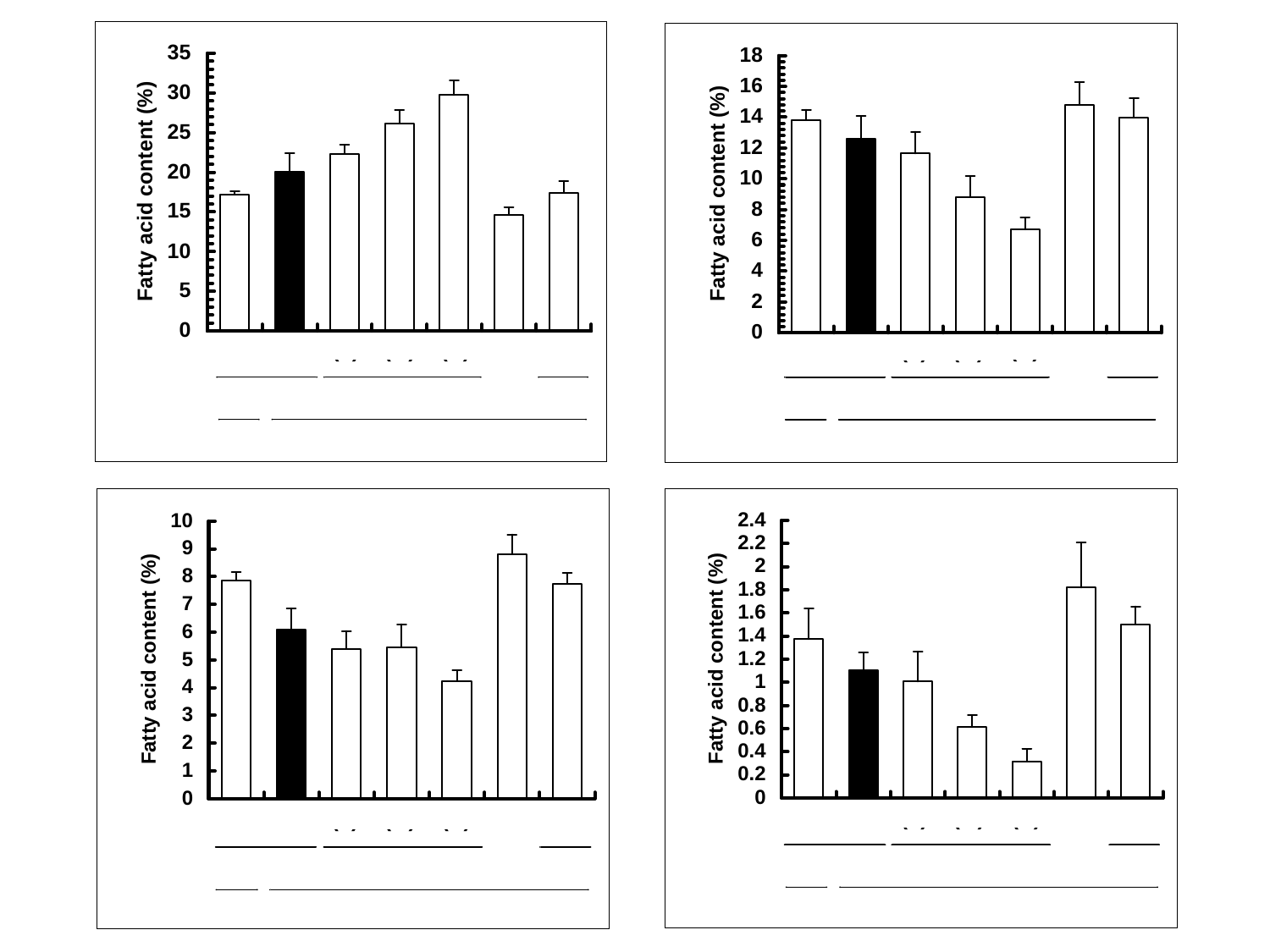

Supplement: Supplementary file 2 — Additional file 2: Figure S1: Fatty acid content of phospholipids ((a) ARA, (b) LA, (c) DHA, and (d) EPA) in the plasma from adjuvant-induced arthritis rats. Data are means ± SD. *p < 0.05, **p < 0.01 versus ADV+/CON diet group (n = 10 per group). (PPT 74 KB) [file 12944_2014_1210_MOESM2_ESM.ppt]

## Slide 1
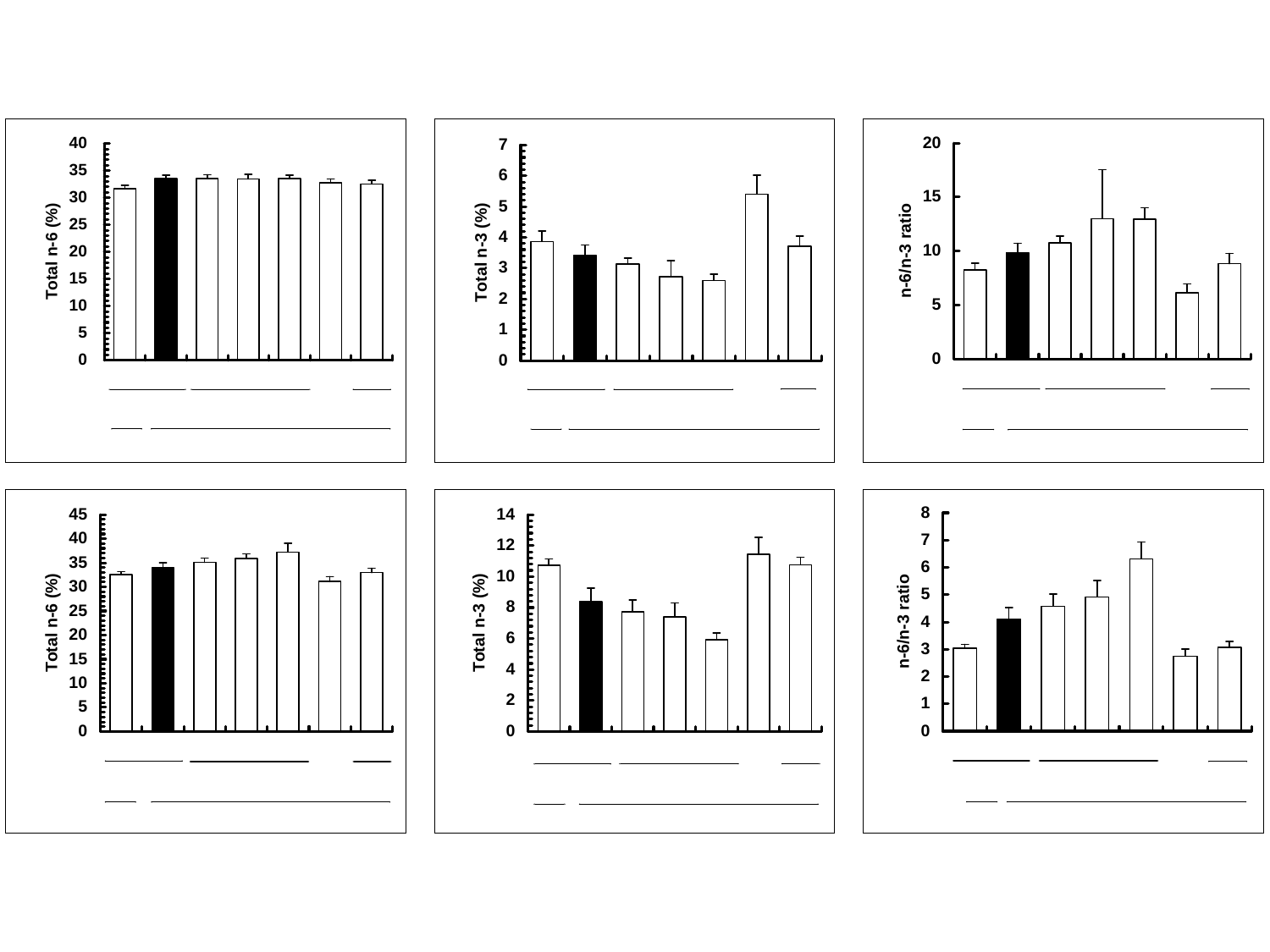

Supplement: Supplementary file 3 — Additional file 3: Figure S2: Total n-6, total n-3 and n-6/n-3 ratio on fatty acid content of phospholipids in the paw (a, b, c) or plasma (d, e, f) from adjuvant-induced arthritis rats. Data are means ± SD. *p < 0.05, **p < 0.01 versus ADV+/CON diet group (n = 10 per group). (PPT 77 KB) [file 12944_2014_1210_MOESM3_ESM.ppt]

## Slide 1
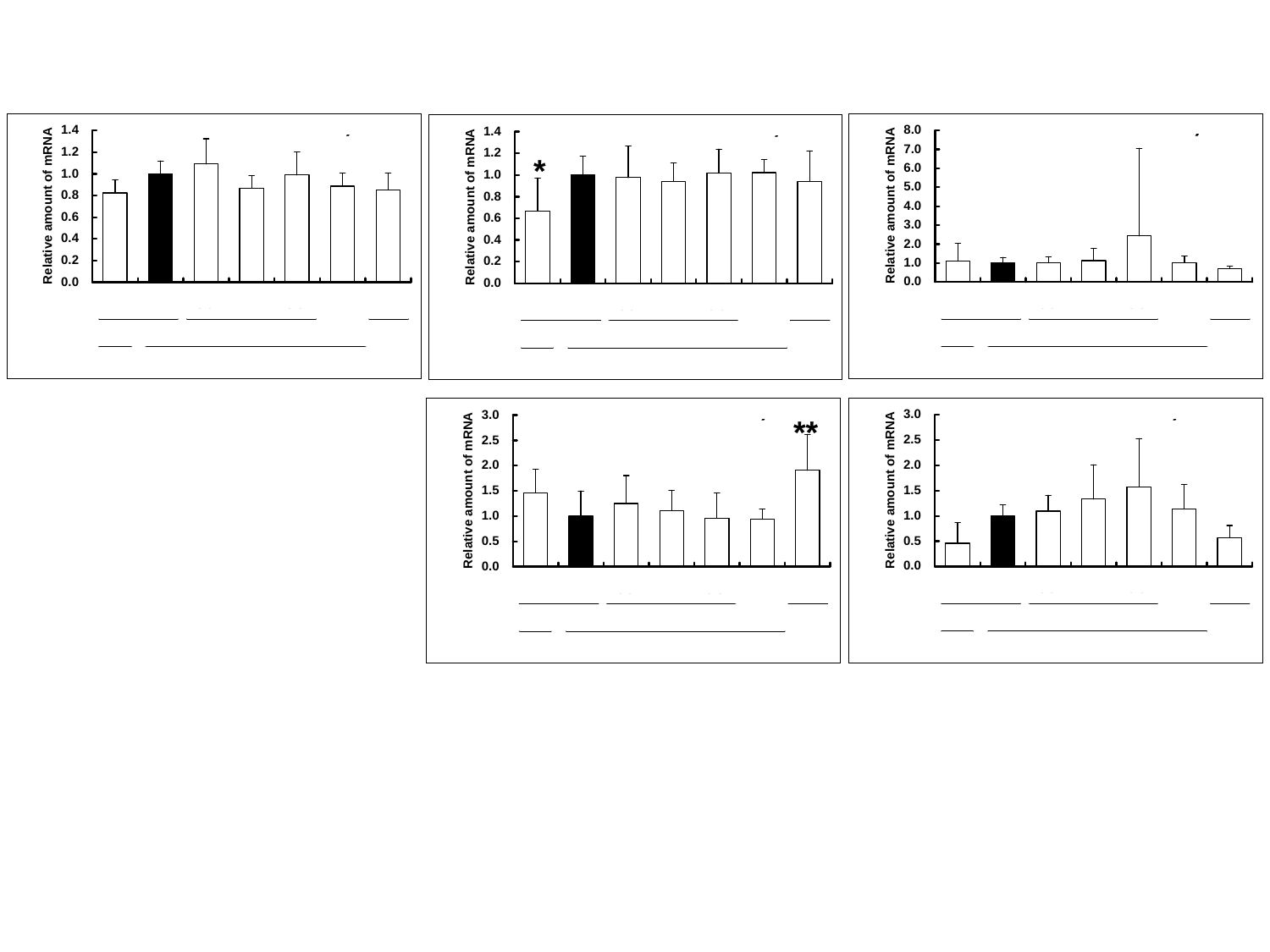

*
**

Supplement: Supplementary file 5 — Additional file 5: Figure S4: Expression of genes related to lipid mediator formation. cPLA2 (a), COX-1 (b), COX-2 (c), 5-LOX (d) and 15-LOX (e) in the ipsilateral hind paw from adjuvant-induced arthritis rats fed CON, ARA(L), ARA(M), ARA(H) and DHA diet. Data are means ± SD. **p < 0.01 versus ADV+/CON diet group (n = 10 per group). (PPT 88 KB) [file 12944_2014_1210_MOESM5_ESM.ppt]

## Slide 1
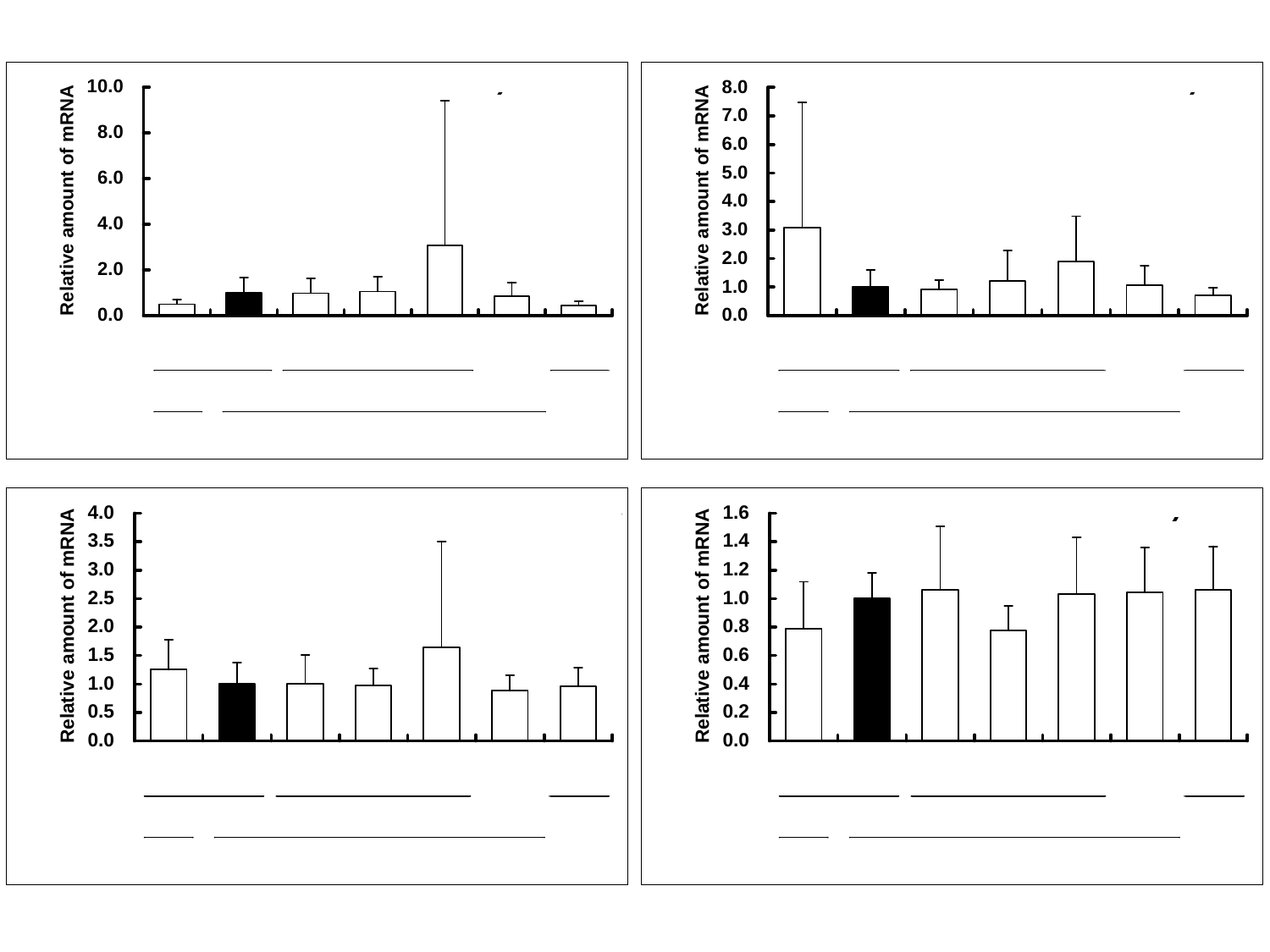

Supplement: Supplementary file 6 — Additional file 6: Figure S5: Expression of genes related to inflammation. IL-1beta (a), IL-6 (b), TNF-α (c), and IL-10 (d) in the ipsilateral hind paw from adjuvant-induced arthritis rats fed a CON, ARA(L), ARA(M), ARA(H), and DHA diet (n = 10 per group). Data are means ± SD. (PPT 75 KB) [file 12944_2014_1210_MOESM6_ESM.ppt]
