# Supplementary material for: Dietary supplementation with arachidonic acid increases arachidonic acid content in paw, but does not affect arthritis severity or prostaglandin E2 content in rat adjuvant-induced arthritis model
Source: Lipids Health Dis. 2015 Jan 16;14:3. doi: 10.1186/1476-511X-14-3 (PMC4417218; doi:10.1186/1476-511X-14-3)
Supplement: Supplementary file 4 — Additional file 4: Figure S3: Body weight change before and after arthritis induction. Rats were fed three types of ARA diet (ARA(L), ARA(M), or ARA(H)), DHA diet, or control diet (CON) from day 0. Adjuvant arthritis was induced by subcutaneous injection of adjuvant containing 0.05 mg of M. butyricum (ADV+) or without M. butyricum (ADV-) on day 29. The IM group rats were orally administered indomethacin (1mgkg−1) once daily from day 29 to day 56. Data are means ± SD. **p < 0.01 versus ADV+/CON diet group (n = 10 per group). (PPT 75 KB) [file 12944_2014_1210_MOESM4_ESM.ppt]

## Slide 1
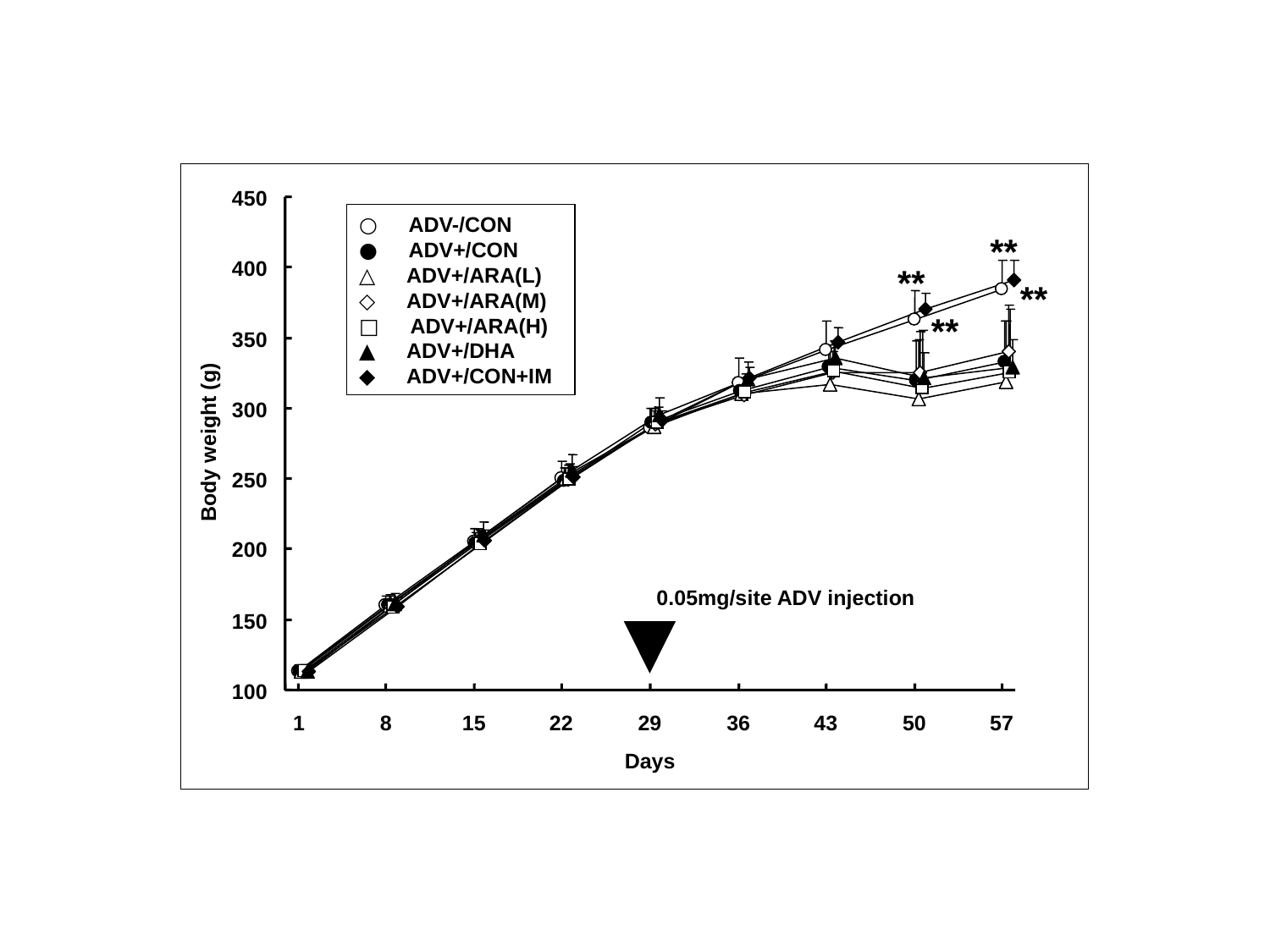

450
○　ADV-/CON
●　ADV+/CON
△　ADV+/ARA(L)
◇　ADV+/ARA(M)
□　ADV+/ARA(H)
▲　ADV+/DHA
◆　ADV+/CON+IM
**
**
400
**
**
350
300
Body weight (g)
250
200
0.05mg/site ADV injection
150
100
1
8
15
22
29
36
43
50
57
Days
